# Association Between Race, Ethnicity, and Sex With Colorectal Cancer Screening Use Among US Adults With and Without Self-Reported Depressive Disorders, Behavioral Risk Factor Surveillance System, 2022

**DOI:** 10.5888/pcd23.250296

**Published:** 2026-04-16

**Authors:** Blanca Muñoz Villareal, Natalia Nielsen, Yiyang Yuan, Maira Castañeda-Avila

**Affiliations:** 1 Division of Epidemiology, Department of Population and Quantitative Health Sciences, University of Massachusetts Chan Medical School, Worcester, Massachusetts

## Abstract

**Introduction:**

Colorectal cancer (CRC) is the third leading cause of cancer death in men and the fourth in women in the US. Racial, ethnic, and sex disparities persist, and people with depressive disorders may delay or skip CRC screening, contributing to later-stage diagnosis and worse outcomes. We examined the association between sex, race, ethnicity, and CRC screening by depression history.

**Methods:**

We analyzed 2022 Behavioral Risk Factor Surveillance System data for adults aged 45 to 74 years (n = 222,601), classifying CRC screening status as up to date or not up to date/never screened. The main predictors were sex, race, and ethnicity (eg, non-Hispanic Black women) and self-reported depression. We used Poisson regression to estimate adjusted prevalence ratios (aPRs).

**Results:**

The weighted sample represented approximately 108 million United States adults aged 45 to 74 years. People with depression were slightly more likely to be up to date with CRC screening (aPR = 1.05; 95% CI, 1.04–1.07) compared with those without depression. Asian (aPR = 0.77; 95% CI, 0.72–0.83), American Indian or Alaska Native (aPR = 0.81; 95% CI, 0.75–0.88), and Hispanic (aPR = 0.83; 95% CI, 0.80–0.86) people were less likely to be up to date compared with White people. After adjusting for confounders, we found that depression was associated with increased screening only among White women, who were 4% more likely to be up to date (aPR = 1.04; 95% CI, 1.01–1.07) than White men with depression.

**Conclusion:**

Outreach programs should aim to increase awareness of and accessibility to CRC screening among people who are at high-risk for CRC, such as Hispanic and Asian populations. More research is needed to better understand how depression and its symptoms influence CRC screening use.

**Summary**

**What is already known on this topic?**

Racial and ethnic disparities in colorectal cancer (CRC) screening are well documented. Findings related to sex and depression have been inconsistent.

**What is added by this report?**

We examined combined racial, ethnic, and sex groups (eg, Hispanic women) and stratified by self-reported depression. Asian, American Indian or Alaska Native, and Hispanic people were less likely to be up to date, regardless of sex or depression status. Only White women with depression had slightly higher screening rates than White men.

**What are the implications for public health practice?**

Interventions are needed for racial and ethnic minorities. Further research is warranted to understand the role of depression in CRC screening.

## Introduction

Colorectal cancer (CRC) is the third most common diagnosis and the third leading cause of cancer death for men and the fourth for women in the US (1). CRC screening reduces incidence and mortality rates (2); however, disparities exist. Asian and Hispanic people have lower screening rates than White people, while Black people tend to have similar screening rates as their White counterparts (3). Women generally participate in CRC screening programs more than men, but this statistic varies globally due to sociodemographic and contextual factors that are influenced by gender (4). Studies examining the CRC screening use among patients with mental health issues have yielded inconsistent findings (5–10). Understanding of the complex interplay between depression history, race, ethnicity, and sex with the use of CRC screening is limited. Disparities in sex, race, and ethnicity can affect CRC screening behaviors, and depression status adds complexities (11–14). Depression may influence CRC screening through stigma, reduced motivation, and differential health care engagement (15–17).

Despite existing research on disparities in CRC screening, substantial gaps remain in understanding the relationship between race, ethnicity, sex, and depression status. Previous studies have focused on individual factors rather than their combined effects, which limits our understanding of how these elements influence CRC screening behaviors. We examined the association between sex, race, and ethnicity and CRC screening by depression history. We hypothesized that CRC screening use would vary across racial, ethnic, and sex groups and that the magnitude and pattern of these differences would differ by depression status. By identifying population subgroups who experience multiple barriers to being screened, such as racial and ethnic minority men with depression, these findings may inform the design of tailored interventions, including culturally responsive screening outreach, integration of CRC screening reminders within mental health care settings, and specific patient navigation strategies aimed at reducing structural and psychosocial barriers to screening.

## Methods

### Study design and sample

We used cross-sectional data from the 2022 Behavioral Risk Factor Surveillance System (BRFSS), a health-related survey of the US population (18). The BRFSS collects data from US residents concerning their health-related risk behaviors, chronic health conditions, and use of preventive services (18,19). Data are collected annually in all 50 states, the District of Columbia, Guam, Puerto Rico, and the US Virgin Islands (18). The median response rate for the 2022 BRFSS survey was 45% (range, 22.8%–66.8%) (20). For the 2022 survey, BRFSS collected health information from the noninstitutionalized population of US adults aged 18 years or older (18).

The US Preventive Services Task Force (USPSTF) recommends CRC screening starting at age 45 years (21). In this study, we included participants who were US adults aged 45 to 74 years. Of the 445,132 participants participating in the 2022 BRFSS, we excluded participants outside of the age range for recommended CRC screening (aged <45 years or >74 years; n = 211,054). Additionally, we excluded participants who responded with “don’t know/not sure” or “refused” or who had missing responses for self-reported depression (n = 1,080), health insurance (n = 6,225), and employment status (n = 4,172). The final study population was 222,601 people, of which 119,476 were women and 103,125 were men. Health insurance and employment status were included as covariates in the multivariable models; therefore, participants with missing data on these variables were excluded to permit complete-case analysis.

### Study variables

**Colorectal cancer screening. **We defined CRC screening status based on the USPSTF recommendations, using data from the BRFSS survey, which asked about various screening methods: stool tests, sigmoidoscopy, colonoscopy, stool DNA tests, virtual colonoscopy, and sigmoidoscopy combined with a blood stool test. We considered respondents up to date with CRC screening if they had completed one of these tests within the USPSTF-specified timeframes: a blood stool test within the past year, sigmoidoscopy within the past 5 years, colonoscopy within the past 10 years, a stool DNA test within the past 3 years, virtual colonoscopy within the past 5 years, or both a sigmoidoscopy within the past 10 years and a blood stool test within the past year. We classified respondents’ screening status as either up to date or not up to date/never screened based on their self-reported timing of CRC screening tests.

**Groups jointly defined by race, ethnicity, and sex. **We created a composite variable for race, ethnicity, and sex to examine intersectional differences in CRC screening use. Rather than being stratified by sex alone, this approach allows for the simultaneous assessment of how race, ethnicity, and sex jointly shape access to and use of preventive health services, reflecting sociocultural positioning rather than biologic differences. The racial and ethnic categories were American Indian or Alaska Native (AI/AN), non-Hispanic; Asian, non-Hispanic; Black, non-Hispanic; Hispanic; White, non-Hispanic; and Other, non-Hispanic (including Native Hawaiian or Other Pacific Islander, non-Hispanic, and Multiracial, non-Hispanic). The sex categories were female and male. We combined these variables to create 12 intersecting race, ethnicity, and sex groups: Asian female, Asian male, AI/AN female, AI/AN male, Hispanic female, Hispanic male, Black female, Black male, Other race female, Other race male, White female, and White male. This intersectional approach allows identification of compounded disparities that may be obscured in single-axis analyses.

**Self-reported depression history.** We determined self-reported depression history based on responses to the question, “Have you ever been told by a doctor or other health professional that you have a depressive disorder (including depression, major depression, dysthymia, or minor depression)?” Responses were recorded as yes or no, and the examples listed in the question stem were not collected as separate categories; therefore, depression history was treated as a binary variable.

**Covariates.** We analyzed potential confounders for their impact on the association between race, ethnicity, sex, self-reported depressive disorders, and being up to date with CRC screening. The covariates included in the adjusted models were selected based on theoretical relevance and availability across the 2022 BRFSS data set. These covariates were age (45–64 y or 65–74 y), with categories chosen to reflect differences in CRC screening eligibility and health care use before and after Medicare eligibility at age 65; annual income (<$35,000 or ≥$35,000), categorized to distinguish lower-income adults who may face more financial barriers to preventive care; education (some college or more vs high school graduate or less); marital status (married vs unmarried), where unmarried included divorced, widowed, separated, and never married respondents; employment status (employed, homemaker or student, unemployed or unable to work, or retired); home ownership status (rent home or other arrangements vs own); insurance status (yes or no); having had a routine checkup within the past 12 months (yes or no); having a personal doctor (yes or no); inability to afford a doctor in the past year (yes or no); number of chronic conditions (0 or 1 vs ≥2); days without good mental health in the past 30 days (continuous); and days the poor physical or mental health kept the respondent from doing their usual activities in the past 30 days (continuous).

### Data analysis

We described and compared the characteristics of participants who were up to date with CRC screening with those who were not up to date or had never been screened. We used Poisson models with robust variance estimation to directly estimate prevalence ratios (PRs) and 95% CIs, an approach recommended for cross-sectional analyses of common binary outcomes, where odds ratios from logistic regression may overestimate associations (22). Robust variance estimation was used to account for potential overdispersion and departures from the Poisson assumption of equal mean and variance. We first estimated crude (unadjusted) PRs for the association between CRC screening and each exposure of interest. We then estimated adjusted PRs (aPRs) by using multivariable models. We evaluated the association between CRC screening and jointly defined race, ethnicity, and sex groups, stratified by depression history with White men as the reference group.

All potential covariates were initially entered into multivariable models, and their effect on the association of interest was assessed by using a change-in-estimate criterion. The final set of variables for statistical adjustment was selected based on clinical judgment and a change in the effect estimate of higher than 10% from univariate analysis. Although multiple covariates were considered, only age, health insurance, and employment status met the criteria for inclusion in the final adjusted models. We estimated crude and aPRs for the association between CRC screening and 1) depression history, 2) sex, 3) race and ethnicity, and 4) jointly defined race ethnicity, and sex groups. All analyses incorporated BRFSS sampling weights to account for the complex survey design, including unequal probabilities of selection, nonresponse, and poststratification adjustments. Weighting variables provided by the Centers for Disease Control and Prevention were applied to produce nationally representative estimates of US adults aged 45 to 74 years. We conducted the analysis by using STATA version MP 18.0 (StataCorp LLC).

## Results

The unweighted total sample size for this study was 222,601 people. After applying BRFSS weights, our weighted sample represented approximately 108 million adults aged 45 to 74 years. Among adults who were never screened or not up to date with CRC screening, 80.7% were aged 45 to 64, 6.7% were Asian, 18.8% were Hispanic, 61.5% were employed, 25% rented their home, 10.6% did not have health insurance, 27.8% had never had or had not had a routine checkup in the past 12 months, 17.3% did not have a personal doctor, and 12.8% reported being unable to afford seeing a doctor. Among adults who were up to date with CRC screening, 38.1% were aged 65 to 74, 69.4% were White, 67.5% were married, and 34.7% were retired (Table 1).

**Table 1:** Sociodemographic Characteristics of Adults Aged 45–74 Years (N = 222,601), by Colorectal Cancer Screening Status, Behavioral Risk Factor Surveillance System, 2022^a^

Characteristic	Never/not up to date	Up to date
**Unweighted, n (%)**	78,675 (35.3)	143,926 (64.7)
**Weighted, n (%)**	42,851,423 (39.5)	65,515,659 (60.5)
**Age, %, y**		
45–64	80.7	61.9
65–74	19.3	38.1
**Women, %**	50.6	52.6
**Race and ethnicity, %**		
American Indian or Alaska Native	1.6	1.1
Asian	6.7	3.9
Black	11.6	12.0
Hispanic	18.8	10.9
Other race^b^	3.1	2.8
White	58.1	69.4
**Annual income <$35,000, %**	33.0	24.7
**High school graduate or less, %**	42.2	31.8
**Married, %**	61.1	67.5
**Employment status, %**		
Employed	61.5	49.1
Homemaker or student	5.7	3.7
Unemployed or unable to work	15.8	12.5
Retired	17.0	34.7
**Rent home or other arrangements, %**	25.0	13.6
**No health insurance, %**	10.6	2.0
**Time since routine checkup >12 months, %**	27.8	10.0
**No personal doctor, %**	17.3	4.6
**Inability to afford a doctor in the past year, %**	12.8	5.9
**Two or more chronic conditions^c^, %**	47.0	58.3
**Days without good mental health in past 30 days, mean (SD)**	4.4 (8.7)	3.8 (8.0)
**Days that poor physical or mental health prevented patient from doing usual activities in past 30 days, mean (SD)**	6.2 (9.9)	5.9 (9.6)

Abbreviation: SD, standard deviation.

a Data for the following variables were missing: income (n = 31,656), education (n = 463), marital status (n = 1,130), home ownership (n = 948), time since routine checkup (n = 1,677), personal doctor (n = 1,215), medical cost (n = 533), days without good mental health (n = 3,699), and mental health status (n = 102,903).

b Other race includes Native Hawaiian or Other Pacific Islander or Multiracial.

c Includes arthritis, asthma, binge drinking, cancer, cardiovascular disease, chronic obstructive pulmonary disease, depression, diabetes, kidney disease, obesity, skin cancer, and smoking.

After adjusting for confounders, people with self-reported depression had a slightly higher likelihood of being up to date with CRC screening (aPR = 1.05; 95% CI, 1.04–1.07) compared with those without depression (Table 2). No difference was observed by sex (aPR = 1.01; 95% CI, 1.00–1.03). Asian (aPR = 0.77; 95% CI, 0.72–0.83), AI/AN (aPR = 0.81; 95% CI, 0.75–0.88), and Hispanic (aPR = 0.83; 95% CI, 0.80–0.86) people were less likely to be up to date with CRC screening. In terms of race, ethnicity, and sex combinations, aPRs were generally similar within each racial and ethnic group. However, AI/AN, Hispanic, and Other race groups showed slight differences by sex. Specifically, within most racial and ethnic groups, women were slightly more likely to be up to date with CRC screening than men. When compared with the reference group of White men, AI/AN women were 16% less likely to be up to date with CRC screening, whereas AI/AN men were 21% less likely to be up to date with CRC screening, indicating a modest within-group sex difference despite lower screening rates overall.

**Table 2:** Association Between Depression and Race, Ethnicity, and Sex With Colorectal Cancer Screening Among Adults Aged 45–74 Years (N = 222,601), Behavioral Risk Factor Surveillance System, 2022

Model	Crude prevalence ratio (95% CI)	Adjusted prevalence ratio (95% CI)^a^
**Model 1: depression and colorectal cancer screening^b^**		
Depression		
Yes	1.06 (1.04–1.08)	1.05 (1.04–1.07)
No	1 [Reference]	1 [Reference]
**Model 2: sex and colorectal cancer screening^c^**		
Sex		
Female	1.03 (1.02–1.05)	1.01 (1.00–1.03)
Male	1 [Reference]	1 [Reference]
**Model 3: race and ethnicity and colorectal cancer screening^d^**		
Asian	0.73 (0.68–0.78)	0.77 (0.72–0.83)
AI/AN	0.78 (0.71–0.85)	0.81 (0.75–0.88)
Hispanic	0.73 (0.70–0.75)	0.83 (0.80–0.86)
Other	0.89 (0.85–0.94)	0.93 (0.88–0.98)
Black	0.95 (0.92–0.97)	0.98 (0.96–1.00)
White	1 [Reference]	1 [Reference]
**Model 4: race, ethnicity, and sex and colorectal cancer screening^e^**		
Asian female	0.73 (0.65–0.81)	0.77 (0.68–0.86)
Asian male	0.74 (0.67–0.81)	0.78 (0.71–0.85)
AI/AN female	0.82 (0.74–0.91)	0.84 (0.76–0.94)
AI/AN male	0.75 (0.65–0.87)	0.79 (0.69–0.90)
Hispanic female	0.76 (0.73–0.80)	0.85 (0.81–0.90)
Hispanic male	0.70 (0.66–0.74)	0.80 (0.76–0.84)
Other race female	0.94 (0.87–1.01)	0.96 (0.89–1.03)
Other race male	0.86 (0.79–0.93)	0.91 (0.84–0.99)
Black female	0.98 (0.95–1.00)	0.99 (0.97–1.02)
Black male	0.93 (0.89–0.96)	0.97 (0.93–1.00)
White female	1.02 (1.00–1.03)	1.00 (0.99–1.02)
White male	1 [Reference]	1 [Reference]

Abbreviation: AI/AN, American Indian or Alaska Native.

a Wald test for interaction between depression and sex, as well as depression and race and ethnicity; no significant differences found (*P* < .05).

b Model 1 adjusted for race, ethnicity, sex, age, health insurance, and employment status.

c Model 2 adjusted for depression, race, ethnicity, age, health insurance, and employment status.

d Model 3 adjusted for depression, sex, age, health insurance, and employment status.

e Model 4 adjusted for depression, age, health insurance, and employment status.

**Figure 1:**
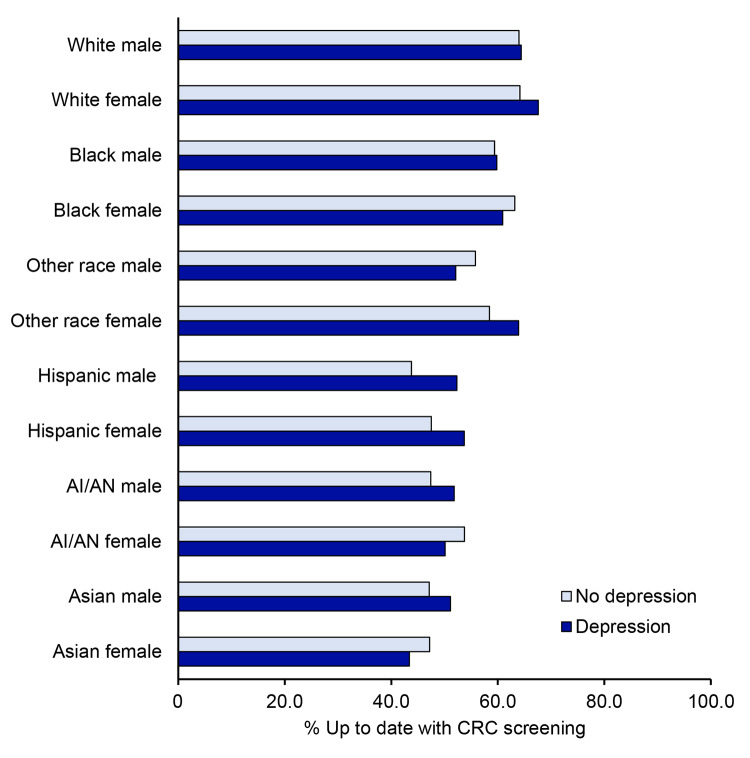
Percentage of respondents up to date with colorectal cancer screening, by race, ethnicity, sex, and depression, adults aged 45–74 years (N = 222,601), Behavioral Risk Factor Surveillance System, 2022. Abbreviations: AI/AN, American Indian or Alaska Native; CRC, colorectal cancer.

**Figure 2:**
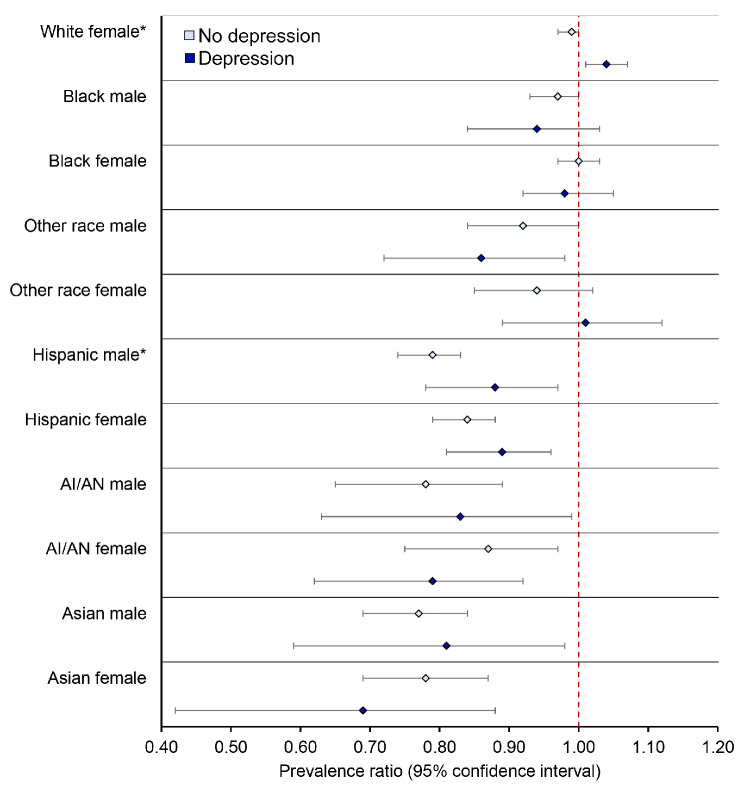
Association between race/ethnicity/sex with colorectal cancer screening, stratified by depression, among adults aged 45–74 years (N = 222,601), Behavioral Risk Factor Surveillance System, 2022. White men are the reference group. Asterisks indicate significance. Abbreviation: AI/AN, American Indian/Alaska Native.

Overall, people with self-reported depression had higher screening rates, although there were some exceptions. For instance, Black women (63.2% vs 60.9%), Other race men (55.8% vs 52.1%), AI/AN women (53.7% vs 50.1%), and Asian women (47.2% vs 43.4%) without depression were slightly more likely to be up to date with CRC screening than those with depression (Figure 1). Adjusted prevalence ratios for the association between groups jointly defined by race/ethnicity and sex with CRC screening, stratified by depression history, are presented in Figure 2 and Table 3. White people were generally more likely to be up to date with CRC screening (Table 3). In contrast, Asian, AI/AN, and Hispanic men and women, regardless of depression history, were less likely to be up to date compared with White men. For example, among people with self-reported depression, Asian women had an aPR of 0.69 (95% CI, 0.50–0.96), and AI/AN women had an aPR of 0.79 (95% CI, 0.66–0.96), indicating significantly lower screening rates compared with White men. Depression appeared to have an effect only among White women. White women with depression were 4% more likely (aPR = 1.04; 95% CI, 1.01–1.07) to be up to date with screening, while no difference was observed among White women without depression (Figure 2 and Table 3).

**Table:** 

Category	Depression, %	No depression, %
Asian female	43.4	47.2
Asian male	51.1	47.1
AI/AN female	50.1	53.7
AI/AN male	51.8	47.4
Hispanic female	53.7	47.5
Hispanic male	52.3	43.8
Other race female	63.9	58.4
Other race male	52.1	55.8
Black female	60.9	63.2
Black male	59.8	59.4
White female*	67.6	64.1
White male	64.4	64.0

**Table:** 

Category	No depression	Depression
	Prevalence ratio (95% CI)	
Asian female	0.78 (0.69–0.87)	0.69 (0.50–0.96)
Asian male	0.77 (0.70–0.85)	0.81 (0.64–1.03)
AI/AN female	0.87 (0.77–0.99)	0.79 (0.66–0.96)
AI/AN male	0.78 (0.67–0.91)	0.83 (0.67–1.03)
Hispanic female	0.84 (0.80–0.89)	0.89 (0.82–0.97)
Hispanic male*	0.79 (0.75–0.84)	0.88 (0.79–0.98)
Other race female	0.94 (0.86–1.03)	1.01 (0.90–1.13)
Other race male	0.92 (0.84–1.00)	0.86 (0.74–1.00)
Black female	1.00 (0.97–1.03)	0.98 (0.91–1.04)
Black male	0.97 (0.94–1.01)	0.94 (0.85–1.04)
White female*	0.99 (0.98–1.01)	1.04 (1.01–1.07)

**Table 3:** Association Between Race, Ethnicity, and Sex With Colorectal Cancer Screening, Stratified by Self-Reported Depression, Adults Aged 45–74 Years (N = 222,601), Behavioral Risk Factor Surveillance System, 2022

Category	Crude prevalence ratio (95% CI)		Adjusted prevalence ratio^a^ (95% CI)	
	Depression	No depression	Depression	No depression
Asian female	0.67 (0.48–0.94)	0.74 (0.66–0.83)	0.69 (0.50–0.96)	0.78 (0.69–0.87)
Asian male	0.79 (0.59–1.07)	0.74 (0.67–0.81)	0.81 (0.64–1.03)	0.77 (0.70–0.85)
AI/AN female	0.78 (0.65–0.93)	0.84 (0.74–0.95)	0.79 (0.66–0.96)	0.87 (0.77–0.99)
AI/AN male	0.80 (0.65–0.99)	0.74 (0.63–0.87)	0.83 (0.67–1.03)	0.78 (0.67–0.91)
Hispanic female	0.83 (0.76–0.91)	0.74 (0.70–0.79)	0.89 (0.82–0.97)	0.84 (0.80–0.89)
Hispanic male	0.81 (0.72–0.91)	0.68 (0.64–0.72)	0.88 (0.79–0.98)	0.79 (0.75–0.84)
Other race female	0.99 (0.88–1.12)	0.91 (0.83–1.00)	1.01 (0.90–1.13)	0.94 (0.86–1.03)
Other race male	0.81 (0.69–0.95)	0.87 (0.80–0.95)	0.86 (0.74–1.00)	0.92 (0.84–1.00)
Black female	0.94 (0.88–1.01)	0.99 (0.96–1.02)	0.98 (0.91–1.04)	1.00 (0.97–1.03)
Black male	0.93 (0.83–1.04)	0.93 (0.89–0.97)	0.94 (0.85–1.04)	0.97 (0.94–1.01)
White female	1.05 (1.02–1.08)	1.00 (0.99–1.02)	1.04 (1.01–1.07)	0.99 (0.98–1.01)
White male	1 [Reference]	1 [Reference]	1 [Reference]	1 [Reference]

Abbreviation: AI/AN, American Indian or Alaska Native.

a Adjusted for age, health insurance, and employment status.

## Discussion

We examined the association between race/ethnicity, and sex with CRC screening, stratified by self-reported depression history. Overall, adults with self-reported depression were slightly more likely to be up to date with CRC screening compared with those without depression. However, within the group reporting depression, this pattern was primarily observed among White people. Specifically, White women with depression were more likely to be up to date than White men with depression. Most other racial and ethnic groups, regardless of depression history, had lower screening rates compared with White men with or without depression.

Consistent with prior studies, we found that Asian, AI/AN, and Hispanic people were significantly less likely to be up to date with CRC screening compared with White people, while Black and Other race people had similar screening rates. These findings align with research using 2018 BRFSS and 2010 and 2015 National Health Interview Survey data, which showed that adjusting for social determinants of health reduced disparities among Hispanic, AI/AN, and Other race groups, but Asian adults continued to exhibit significantly lower screening odds (3,13). For instance, Asian Americans living in ethnically dense neighborhoods were less likely to complete CRC screening even after adjusting for sociodemographic and psychosocial factors, highlighting the influence of structural and community-level barriers (14). Likewise, another study reported persistently low screening among Asian Americans even after accounting for health care access (3). Our findings also align with recent work showing no significant differences in CRC screening between Black and White adults with chronic conditions, suggesting that disparities may be narrowing in some groups (11). Nevertheless, barriers persist for many populations. A systematic review applying the 5As framework identified race and ethnicity, limited English proficiency, cultural perceptions, fear, and mistrust as enduring structural obstacles to screening (10). Although earlier studies in equal-access systems found lower screening among Black patients (23), these gaps may be diminishing and likely vary by context. In our study, we found no major sex-based differences overall, consistent with reports of minimal sex disparities after adjustment (12,13). However, other evidence suggests that women may face distinct challenges in the cancer care continuum, such as delayed diagnosis or different treatment trajectories (4), and some studies have shown slightly higher screening adherence among women in specific subgroups (3).

Compared with non-Hispanic White men who served as the reference group, all other sex, race, or ethnicity combinations had lower screening rates. However, within each racial or ethnic group, women generally had slightly higher screening rates than men. This pattern suggests a modest sex-based advantage among racial and ethnic minority groups. Others have described gender-based disparities without disaggregating by race or ethnicity (4). One recent analysis using BRFSS data demonstrated how race, ethnicity, gender identity, and sexual orientation intersect to shape cancer screening behaviors (24). That study found that transgender women of Asian and Hispanic backgrounds had the lowest CRC screening rates (24). These findings underscore the value of intersectional frameworks in identifying subpopulations, such as racial minority men, who may benefit from tailored outreach strategies.

In examining the role of depression, we found that people with self-reported depressive disorders were slightly more likely to be up to date with CRC screening. This contrasts with a global meta-analysis that documented significantly lower screening rates among people with mental illness, largely due to stigma, fragmented care, and lower health care engagement (6). Depression-related stigma may further complicate interpretation of these findings, particularly among racial and ethnic minority populations, where mental health conditions are more likely to be underreported or untreated. Misclassification of depression in these groups could attenuate observed differences and mask the true extent of mental health–related barriers to screening. Similarly, US veterans with serious mental illness were less likely to receive CRC screening even within an equal-access health system (8). However, several US-based studies reported higher screening rates among people with depression, possibly reflecting more contact with health care providers. For example, people with self-reported depression had higher odds of undergoing screening colonoscopy (9,25). Additional nuance comes from research differentiating by type and severity of mental illness. While people with serious mental illness tend to have lower screening participation, those with mild to moderate conditions may not experience reduced screening (5).

International studies provide context for interpreting these findings. In countries with universal health care and population-based screening programs, such as many in Europe and Japan, depression has been associated with lower screening use (7). These cross-national differences may reflect variations in how health care is accessed and delivered. In the US, insurance coverage can facilitate both the diagnosis of depression and engagement with preventive services. Our analyses adjusted for health insurance status, which helps account for its potential confounding role in the observed association between depression and screening. Nevertheless, it is possible that people with depression captured in BRFSS represent those with greater health care contact, including being diagnosed and managed in clinical settings. As such, some selection may still occur based on help-seeking behaviors or system engagement. Future studies should examine the interplay between mental health, health care use, and insurance incentives in influencing preventive care.

When we stratified CRC screening patterns by depression history, we observed that race, ethnicity, and sex-based disparities remained largely unchanged. One notable exception was among non-Hispanic White adults. White women with depression were significantly more likely to be up to date with screening compared with White men with depression, suggesting a potential interaction between sex and depression in this subgroup. This finding may be due to increased health care engagement among women with depression. Adults from all other racial and ethnic groups, regardless of sex or depression history, remained significantly less likely to be screened than White men. These patterns suggest that while depression may influence health care engagement for some groups, it does not substantially alter entrenched structural barriers to CRC screening faced by racial and ethnic minority populations. This is consistent with previous literature showing that people with mental health conditions often face systemic barriers to care and that these challenges are exacerbated among racial and ethnic minorities (16). For example, an intersectional study of depression care found that racial and ethnic minority women were less likely than White women to be screened or treated for depression, pointing to layered structural inequities (26). Similarly, another study in a safety-net health system found that the intersection of race, ethnicity, language, and mental health status significantly influenced CRC screening use, with people facing multiple barriers being less likely to be up to date (27). Our findings suggest that although depression may increase health care engagement for certain subgroups, such as White women, it does not reduce longstanding disparities in preventive care among populations with less access.

### Strengths and limitations

Our study has several strengths. One is the use of data from the 2022 BRFSS, a large and nationally representative survey of the US population. This study is among the few to comprehensively examine the intersections of race, ethnicity, sex, and depression history in relation to CRC screening use, offering valuable insights into disparities across these dimensions. The large sample size and diverse population enhance the generalizability of our findings in the US, although results may not be applicable to countries with health care systems in which patterns of access and use may vary. Additionally, the use of recent data allows for an up-to-date assessment of CRC screening behaviors. The BRFSS also enables stratified analyses across subpopulations, allowing for a deeper examination of disparities. 

Our study also has limitations. As a cross-sectional analysis, it captured data at a single point in time and cannot assess temporal trends or causality. The reliance on self-reported survey responses introduces potential recall and social desirability bias. Depression history was based solely on a self-reported yes or no response, without consideration of severity or clinical diagnosis. In addition, BRFSS does not collect information on antidepressant medication use or mental health treatment, which may further contribute to misclassification of depression. As prior research suggests, reliance on self-reported diagnosis without treatment indicators may differentially underestimate depression among populations affected by stigma and barriers related to mental health care. This measure may underestimate the true prevalence of depression, particularly among racial and ethnic minority populations where stigma, cultural norms, and barriers to mental health care may reduce diagnosis and disclosure. Consequently, some people classified as not having depression may nonetheless experience depressive symptoms that influence health care use. In addition, although we aimed to include variables related to social determinants of health, these were excluded because of inconsistent availability across states in the 2022 BRFSS. This exclusion may have limited our ability to fully examine the role of structural factors in screening disparities. Finally, the BRFSS may not capture all relevant influences on CRC screening, such as the quality of patient–provider interactions or culturally tailored outreach efforts.

### Conclusion

Our study identified significant disparities in CRC screening use across race, ethnicity, and sex, with some variation by depression history. White women with self-reported depression were more likely to be up to date with CRC screening compared with White men with depression, suggesting potential differences in health care engagement within this subgroup. In contrast, people from racial and ethnic minority groups, regardless of depression history or sex, were consistently less likely to be screened compared with White men. These findings highlight persistent inequities in CRC screening, particularly among Hispanic and Asian populations. Public health efforts should prioritize tailored outreach to these groups to improve awareness and uptake. Although our findings suggest that depression may be associated with increased screening in some populations, more research is needed to better understand the mechanisms behind this relationship and whether it varies by severity, health care access, or sociodemographic context. Addressing the complex interplay of social and demographic factors remains essential for reducing disparities in CRC screening and improving early detection across diverse populations.
